# Measurable residual disease status and outcome of transplant in acute myeloid leukemia in second complete remission: a study by the acute leukemia working party of the EBMT

**DOI:** 10.1038/s41408-021-00479-3

**Published:** 2021-05-12

**Authors:** Maria H. Gilleece, Avichai Shimoni, Myriam Labopin, Stephen Robinson, Dietrich Beelen, Gerard Socié, Ali Unal, Arnold Ganser, Antonin Vitek, Henrik Sengeloev, Ibrahim Yakoub-Agha, Eleni Tholouli, Emmanuelle Polge, Mohamad Mohty, Arnon Nagler

**Affiliations:** 1 Leeds Teaching Hospitals Trust, St James’s University Hospital, Leeds, LS9 7TF United Kingdom; 2 Hematology and Bone Marrow Transplantation Division, Chaim Sheba Medical Center, Tel-Hashomer, Sackler School of Medicine, Tel-Aviv University, 6997801 Tel-Aviv, Israel; 3 EBMT Paris study office/CEREST-TC, Paris, France; 4 University Hospital Bristol NHS Foundation Trust, London, United Kingdom; 5 Department of Bone Marrow Transplantation, West German Cancer Center, University Hospital of Essen, Essen, Germany; 6 Hematologie/Transplantation, Hôpital St Louis, Paris, CEDEX 10 France; 7 Kapadokya BMT Center, Kayseri, Turkey; 8 Department of Hematology, Hemostasis, Oncology and Stem Cell Transplantation, Hannover Medical School, Hannover, Germany; 9 Hematology Service, Institute of Hematology and Blood Transfusion, Prague, Czech Republic; 10 Department of Haematology, Rigshospitalet, Copenhagen Denmark; 11 CHU de Lille, LIRIC, INSER U995, Université de Lille, Lille, France; 12 Manchester Royal Infirmary, Manchester, United Kingdom; 13 Acute Leukemia Working Party, European Society for Blood and Marrow Transplantation Paris Study Office/European Center for Biostatistical and Epidemiological Evaluation in Hematopoietic Cell Therapy (CEREST-TC), Paris, France; 14 Hôpital Saint Antoine, INSERM UMR 938, Paris, France; Université Pierre et Marie Curie, Paris, France; 15 Hematology Division, Chaim Sheba Medical Center, Tel Hashomer, Israel

**Keywords:** Risk factors, Acute myeloid leukaemia

## Abstract

Measurable residual disease (MRD) prior to hematopoietic cell transplant (HCT) for acute myeloid leukemia (AML) in first complete morphological remission (CR1) is an independent predictor of outcome, but few studies address CR2. This analysis by the Acute Leukemia Working Party of the European Society for Blood and Marrow Transplantation registry assessed HCT outcomes by declared MRD status in a cohort of 1042 adult patients with AML CR2 at HCT. Patients were transplanted 2006–2016 from human leukocyte antigen (HLA) matched siblings (*n* = 719) or HLA 10/10 matched unrelated donors (*n* = 293). Conditioning was myeloablative (*n* = 610) or reduced-intensity (*n* = 432) and 566 patients (54%) had in-vivo T cell depletion. At HCT, 749 patients (72%) were MRD negative (MRD NEG) and 293 (28%) were MRD positive (MRD POS). Time from diagnosis to HCT was longer in MRD NEG than MRD POS patients (18 vs. 16 months (*P* < 0.001). Two-year relapse rates were 24% (95% CI, 21–28) and 40% (95% CI, 34–46) in MRD NEG and MRD POS groups (*P* < 0.001), respectively. Leukemia-free survival (LFS) was 57% (53–61) and 46% (40–52%), respectively (*P* = 0.001), but there was no difference in terms of overall survival. Prognostic factors for relapse and LFS were MRD NEG status, good risk cytogenetics, and longer time from diagnosis to HCT. In-vivo T cell depletion predicted relapse.

## Introduction

Relapse of acute myeloid leukemia (AML) following allogeneic hemopoietic cell transplant (HCT) remains a major cause of treatment failure and indicates the presence of persistent subclinical disease, despite morphological complete remission (CR) at the time of transplant^1–4^. AML is a heterogeneous malignancy associated with a wide variety of fusion genes, mutations, and overexpressed genes^5–7^. Multiple techniques such as multi-parameter flow cytometry immunophenotyping, real-time quantitative polymerase chain reactions, or high throughput sequencing are available to detect so-called “measurable residual disease” (MRD) in the presence of morphological CR^8,9^. Multi-parameter flow cytometry immunophenotyping MRD assays are applicable to 90% of patients with AML and may detect cells with a leukemia-associated immunophenotype or a “different from normal” immunophenotype at a sensitivity of 10^−3^ to 10^−5^ in bone marrow^5,10–15^. In addition, up to 60% of young adults have a molecular marker detectable by real-time quantitative polymerase chain reactions assays and most cases of AML are amenable to molecular tracking by high throughput sequencing assays with sensitivity 10^−4^ to 10^−6^ ^3^. Thus MRD assessments may be used to assess the kinetics of response to therapy, as well as impending relapse, and have become an integral part of present-day clinical trials in AML^3,16^.

MRD status after induction therapy is prognostic of outcome in AML independent of other accepted risk parameters^6,10,12,14,16–26^. Furthermore, multiple large studies have demonstrated the prognostic importance of MRD pre-HCT in CR1 terms of subsequent relapse incidence (RI), leukemia-free survival (LFS), and overall survival (OS)^6,14,27–35^. A previous study undertaken by the European Society for Blood and Marrow Transplantation (EBMT) found that patients with MRD at HCT had inferior survival at 2 years after HCT compared to those with an MRD negative status (56.2% vs. 70% in adults aged less than 50 years and 50.7% vs. 62.1% in patients 50 years and older)^35^.

HCT is usually deferred for patients with AML in CR1 if the relapse risk is less than 35% although only a minority of relapsing patients will achieve CR2 and proceed to HCT^4,36–39^. Breems et al. identified that the duration of CR1, age at relapse, cytogenetic risk factor at diagnosis, and a prior allogeneic HCT could be used to predict the likelihood of CR2^40^. These observations are consistent with other large studies^41–45^.

Those patients who proceed to HCT in CR2 have similar outcomes to patients transplanted in CR1 with a reported survival of 58.2% at 2 years after HCT^45^. Walter et al showed as part of a sub-set analysis that in 70 patients with AML in CR2 treated with myeloablative (MAC) HCT, 3 year OS post-HCT was 73% in MRD negative (MRD NEG) vs. 44% in MRD positive patients (MRD POS)^31^. In the first large series to address the impact of MRD status in AML CR2, we have analyzed the results of allogeneic HCT utilizing a large cohort of patients for whom MRD data had been deposited in the registry of the EBMT.

## Methods

### Study design and data collection

The Acute Leukemia Working Party of the EBMT approved and conducted this study. The EBMT supports data registration from more than 600 transplant centers, predominantly located within Europe. Centers are required to report all HCT with subsequent annual follow-up. EBMT Med A/B standardized data collection forms are completed and submitted to the registry by transplant center personnel following written informed consent from patients in accordance with Center ethical research guidelines^46^. Accuracy of data is assured by the individual transplant centers and by quality control measures such as regular internal and external audits. Since January 1, 2003, all transplant centers have been required to obtain written informed consent prior to data registration with the EBMT, following the Helsinki Declaration of 1975.

The objectives of the study were to assess the impact of MRD status on transplant outcomes in patients with AML CR2 at the time of transplant.

### Eligibility criteria

Eligibility criteria were age ≥18 years, first allogeneic HCT 2006–2016, a diagnosis of de novo AML in CR2 (excluding acute promyelocytic leukemia), and availability of MRD status prior to HCT as declared by the center. A recent survey of EMBT centers indicated that most used a combination of validated MRD assays as directed by the presence of specific mutations detectable by PCR and/or leukemia profiles amenable to detection by flow cytometry^47^. Cytogenetic status was classified using MRC UK criteria while any identified molecular markers at diagnosis were also noted^48^. Donors were restricted to a human leukocyte antigen (HLA) matched sibling donor (MSD) or volunteer unrelated donor with HLA match 10/10 (MUD). The graft source included peripheral blood stem cells (PBSC) or bone marrow grafts. Engraftment was assessed by conventional EBMT standards^46^. The intensity of conditioning and chronic GVHD were classified in accordance with published criteria^49–52^.

Patient, disease, and transplant-related characteristics for the two cohorts (MRD POS/ MRD NEG) were compared by using *χ*^2^ statistics for categorical variables and the Mann-Whitney test for continuous variables. The primary endpoint was leukemia-free survival (LFS). Secondary endpoints were relapse incidence (RI), non-relapse mortality (NRM), OS, acute graft-vs.-host disease (aGVHD), and chronic graft-vs.-host-disease (cGVHD), GVHD-free/relapse-free survival (GRFS). LFS was defined as survival with no evidence of relapse or progression. Relapse was defined as the presence of 5% bone marrow blasts and/or reappearance of the underlying disease. NRM was defined as death without evidence of relapse or progression. OS was defined as the time from alloSCT to death, regardless of the cause. GRFS was defined as events including grade 3–4 acute GVHD, extensive chronic GVHD, relapse, or death in the first post-HCT year^53^. Cumulative incidence was used to estimate the endpoints of NRM, RI, acute and chronic to accommodate for competing risks. To study acute and chronic GVHD, we considered relapse and death to be competing events. Probabilities of OS, LFS, and GRFS were calculated using the Kaplan–Meier method. Univariate analyses were done using Gray’s test for cumulative incidence functions and the log-rank test for OS, GRFS, and LFS. A Cox proportional hazards model was used for multivariate regression. All variables differing significantly between the 2 groups or factors associated with one outcome in univariate analysis were included in the Cox model. In order to test for a center effect, we introduced a random effect or frailty for each center into the model^54,55^. Results were expressed as the hazard ratio (HR) with a 95% confidence interval (95% CI). All tests were 2-sided. The type I error rate was fixed at 0.05 for the determination of factors associated with time-to-event outcomes. Subgroup analyses were stratified by donor type (MSD or MUD). Statistical analyses were performed with SPSS 24.0 (SPSS Inc, Chicago, IL, USA) and R 3.4.0 (R Core Team (2017). R: A language and environment for statistical computing. R Foundation for Statistical Computing, Vienna, Austria. URL https://www.R-project.org/.)

## Results

### Patient, donor, and transplant characteristics

A total of 1042 patients satisfied the entry inclusion criteria for the study and of these, 293 had evidence of MRD at transplant. Patient and donor characteristics are summarized in Table 1.

**Table 1:** Patient and transplant characteristics.

	MRD negative	MRD positive	Test *p*-value
Number	749	293	
Follow-up (surviving patients): median (range) (IQR)	24.6 (0.5–132.5) (7.7–54.3)	26.9 (0.8–121.5) (8.2–60.4)	0.58
Age at transplant: median (range) (IQR)	49.4 (18–78.1) (37.7–58.8)	49.8 (19.5–72.9) (37.6–58.4)	0.89
Year of transplant: median (range)	2012 (2006–2016)	2012 (2006–2016)	0.31
Time from diagnosis to transplant: median (range) (IQR) m	18.2 (3–200) (13.5–27.1)	15.9 (3–200) (11.6–21.8)	<10^−4^
Male patients	390 (52.07%)	168 (57.34%)	0.13
Female patients	359 (47.93%)	125 (42.66%)	
Donor male	445 (60.22%)	189 (64.73%)	0.18
Donor female	294 (39.78%)	103 (35.27%)	
Missing	10	1	
Female recipients and male recipients of male donors	598 (80.27%)	243 (83.22%)	0.28
Male recipients of female donors	147 (19.73%)	49 (16.78%)	
Missing	4	1	
KPS < 80	25 (3.49%)	8 (2.86%)	0.62
KPS > = 80	691 (96.51%)	272 (97.14%)	
missing	33	13	
KPS < 90	148 (21.08%)	63 (22.74%)	0.57
KPS > = 90	554 (78.92%)	214 (77.26%)	
Missing	47	16	
Patient CMV negative	248 (34.35%)	95 (32.76%)	0.63
Patient CMV positive	474 (65.65%)	195 (67.24%)	
Missing	27	3	
Donor CMV negative	331 (45.84%)	146 (51.59%)	0.10
Donor CMV positive	391 (54.16%)	137 (48.41%)	
Missing	27	10	
CMV donor negative/recipient negative	177 (24.96%)	71 (25.18%)	0.24
CMV donor positive/recipient negative	66 (9.31%)	23 (8.16%)	
CMV donor negative/recipient positive	146 (20.59%)	74 (26.24%)	
CMV donor positive/recipient positive	320 (45.13%)	114 (40.43%)	
Missing	40	11	
Cytogenetics			
Good	193 (48.61%)	102 (53.4%)	0.18
Intermediate	188 (47.36%)	77 (40.31%)	
Adverse	16 (4.03%)	12 (6.28%)	
Missing	352	102	
FLT3 negative	94 (63.09%)	66 (70.21%)	0.25
FLT3 positive	55 (36.91%)	28 (29.79%)	
Missing	600	199	
NPM1 negative	42 (33.6%)	31 (42.47%)	0.21
NPM1 positive	83 (66.4%)	42 (57.53%)	
Missing	624	220	
MSD	376 (50.2%)	134 (45.73%)	0.20
VUD HLA 10/10	373 (49.8%)	159 (54.27%)	
MAC	430 (57.41%)	180 (61.43%)	0.24
RIC	319 (42.59%)	113 (38.57%)	

*CMV* cytomegalovirus, *IQR* interquartile range, *KPS* Karnofsky performance status, *MAC* myeloablative conditioning, *MSD* matched sibling donor, *RIC* reduced-intensity conditioning, *MUD* matched unrelated donor.

When considered as two groups, MRD negative vs. MRD positive, the median age at transplant was 49 years in each. Karnofsky Performance Status and distribution of cytogenetic risk categories were also equivalent. There was a preponderance of male patients and donors overall. CMV serological status of patients was similar and although more CMV sero-negative donors were selected for MRD positive than MRD negative patients, this did not reach statistical significance. HLA-matched siblings were more likely to be selected as donors for MRD negative than MRD positive patients but this was not statistically significant. Both groups of patients were more likely to receive MAC HCT than RIC HCT. Time from diagnosis to transplant was shorter for MRD positive (median 15.9 months) than MRD negative recipients (18.2 months, *P* < 10^−4^) but median follow-up after transplant was similar at just over two years.

Conditioning regimens are listed in Supplementary Table 1. The commonest MAC HCT regimens were based on busulfan or TBI while RIC HCT regimens favored fludarabine, particularly when paired with busulfan or melphalan. GVHD prophylaxis is summarized in Supplementary Table 2 and included in vivo T cell depletion in a majority of transplants with equivalent usage in MRD negative (53%) and MRD positive (60%) recipients (*P* = 0.066). Otherwise, GVHD prophylaxis was based on calcineurin inhibitors with a majority of patients being treated with ciclosporin-based regimens.

### Transplant outcomes

Transplant outcomes for the entire cohort are shown in Supplementary Table 3. At 2-year post-transplant overall survival was 62%, LFS 54%, and GRFS 37% while NRM was 18% and RI 29%. At 100 days post-HCT, aGVHD grade III–IV was reported in 9% while at 2 years post-HCT the cumulative incidence of cGVHD was 41%, extensive in 20%.

The median number of days to engraftment was shorter in MRD negative than MRD positive patients (15 vs. 16 days *P* = 0.001) but in both groups engraftment rates exceeded 98% (*P* = 0.4) (Table 2). Death in MRD positive vs. MRD negative patients was predominantly due to relapse (56% vs. 46%), GVHD (16% vs. 21%), or infection (14% vs. 21%) (Table 2).

**Table 2:** Transplant engraftment and toxicity in MRD negative vs. MRD positive patients.

	MRD negative	MRD positive	Test *p*-value
Engraftment failure	8 (1.07%)	5 (1.72%)	0.40
Engrafted	737 (98.93%)	286 (98.28%)	
Missing	4	2	
Time to neutrophils >0.5 × 10^9^/L median (range) (IQR) d	15 (1–41) (13–18)	16 (8–56) (13–19)	0.00
Missing	15	5	
Acute GVHD			
Grade I	113 (15.48%)	55 (18.9%)	0.13
Grade II	115 (15.75%)	42 (14.43%)	
Grade III	33 (4.52%)	18 (6.19%)	
Grade IV	22 (3.01%)	13 (4.47%)	
Grade unknown	15 (2.05%)	1 (0.34%)	
Absence	432 (59.18%)	162 (55.67%)	
Missing	19	2	
No aGVHD II–IV	545 (76.22%)	217 (74.83%)	0.64
aGVHD II–IV	170 (23.78%)	73 (25.17%)	
Missing	34	3	
No cGVHD	430 (63.52%)	184 (66.43%)	0.39
cGVHD	247 (36.48%)	93 (33.57%)	
Missing	72	16	
Limited	113 (49.78%)	29 (32.58%)	
Extensive	114 (50.22%)	60 (67.42%)	
Missing	20	4	
Causes of death			
*N*	281	120	
Cardiac toxicity	3 (1.16%)	0 (0%)	
Haemorhage	3 (1.16%)	1 (0.88%)	
SOS	4 (1.54%)	1 (0.88%)	
Infection	55 (21.24%)	16 (14.16%)	
IP	2 (0.77%)	4 (3.54%)	
GVHD	54 (20.85%)	18 (15.93%)	
Original disease	118 (45.56%)	63 (55.75%)	
Second malignancy	4 (1.54%)	1 (0.88%)	
Other transplant related	16 (6.18%)	9 (7.96%)	
Missing	22	7	

*GVHD* graft vs. host disease, *MRD* measurable residual disease, *SOS* sinusoidal obstructive syndrome.

In univariate analysis of outcomes at 2-year (Supplementary Table 4), MRD positive status at transplant was associated with excess relapse (40% vs. 24% *P* < 0.001), reduced LFS (46% vs. 57% *P* = 0.001), and worse GRFS (28% vs. 41% *P* < 0.001) but overall equivalent survival at 62% compared to MRD negative patients. NRM and cGVHD rates were greater in MRD NEG than MRD POS (19% vs. 14% and 42% vs. 39%) but these did not reach statistical significance.

Survival and LFS were improved in patients whose characteristics included age less than the median of 49-year, good risk cytogenetics at diagnosis, and longer times from diagnosis to transplant (Supplementary Table 4). Relapse was also influenced by cytogenetic risk category and time to transplant but additional adverse factors were female donors for male recipients. NRM was only significantly affected by increasing patient age. T cell depletion had beneficial effects on day 100 rates of Grade III–IV GVHD, as well as 2-year rates of GRFS, cGVHD, and extensive cGVHD.

### Cox regression multivariate analysis

Detailed outcomes of multivariate analysis are listed in Table 3. MRD Negative status conferred a significantly reduced risk of relapse (HR 0.67 CI 0.44–0.73 *P* < 0.001) and extensive cGVHD (HR 0.57 CI 0.4–0.81 *P* = 0.0020 which resulted in improved LFS (HR 0.76 CI 0.62–0.94 *P* = 0.01) and GRFS (HR 0.69 CI 0.57–0.83 *P* < 0.001) but no change in OS. Other established factors had predictable effects on outcomes. Thus, OS and LFS were both enhanced in patients with good risk cytogenetics or prolonged time interval from diagnosis to transplant. While TCD was associated with improved GRFS, there was no concomitant improvement in OS or LFS, probably due to the increased risk of relapse. The use of unrelated donors or female donors for male recipients increased the risk of aGVHD grades II–IV and extensive cGVHD but TCD reduced the risks of all forms of aGVHD and cGVHD. NRM increased with advancing age.

**Table 3:** Cox regression analysis of transplant outcomes using variables derived from the outcome of univariate analysis or established risk factors.

	Relapse			NRM			LFS		
	HR	CI	*p*	HR	CI	*p*	HR	CI	*p*
MRD negative at transplant	0.57	0.44–0.73	1e−05	1.31	0.91–0.90	0.15	0.76	0.62–0.94	0.01
Age (per 10 years)	0.96	0.86–1.07	0.42	1.33	1.15–1.53	0.00	1.09	0.99–1.19	0.07
RIC vs. MAC	1.03	0.79–1.37	0.82	0.82	0.58–1.17	0.29	0.93	0.75–1.16	0.53
Good risk cytogenetics vs. all others	0.62	0.46–0.83	0.00	1.19	0.83–1.70	0.36	0.79	0.62–0.99	0.04
Time from diagnosis to transplant	0.98	0.97–0.99	<10^−5^	1.00	0.99–1.00	0.34	0.99	0.98–0.99	2e−05
VUD vs. MSD	0.79	0.60–1.04	0.10	1.11	0.77–1.60	0.58	0.90	0.72–1.13	0.39
Female donor to male	0.75	0.52–1.07	0.11	1.30	0.88–1.89	0.20	0.94	0.72–1.22	0.63
In vivo TCD	1.35	1.02–1.79	0.03	0.89	0.62–1.27	0.51	1.13	0.91–1.41	0.27
Patient CMV positive	1.03	0.78–1.36	0.82	1.00	0.71–1.43	0.98	1.01	0.81–1.26	0.9175
Donor CMV positive	0.87	0.66–1.15	0.32	1.39	0.98–1.97	0.06	1.06	0.85–1.31	0.61
Transplant center			0.29			0.16			0.21

	OS			GRFS			AGVHD II–IV		
	HR	CI	*p*	HR	CI	*p*	HR	CI	*p*
MRD negative at transplant	1.00	0.77–1.20	0.70	0.69	0.57–0.83	6e−05	0.91	0.69–1.22	0.52
Age (per 10 years)	1.10	1.00–1.20	0.058	1.05	0.97–1.13	0.25	0.94	0.84–1.06	0.29
RIC vs. MAC	1.00	0.78–1.22	0.83	0.98	0.81–1.18	0.80	0.84	0.62–1.14	0.26
Good risk cytogenetics vs. all others	0.72	0.56–0.92	0.01	0.87	0.71–1.06	0.16	1.04	0.76–1.42	0.80
Time from diagnosis to transplant	0.99	0.980–0.99	0.0	1.00	0.99–1.00	0.26	1.00	1.00–1.01	0.16
VUD vs. MSD	0.95	0.75–1.20	0.66	1.14	0.93–1.40	0.20	2.03	1.46–2.81	2e−05
Female donor to male	1.06	0.76–1.32	0.98	1.08	0.86–1.34	0.55	1.48	1.06–2.07	0.02
In vivo TCD	1.10	0.88–1.38	0.40	0.61	0.51–0.74	<10^−5^	0.62	0.45–0.84	0.00
Patient CMV positive	0.95	0.76–1.20	0.68	1.17	0.97–1.42	0.11	0.99	0.73–1.33	0.94
Donor CMV positive	1.03	0.82–1.30	0.78	1.60	0.88–1.28	0.56	1.17	0.87–1.56	0.30
Transplant center			0.30			0.34			0.15

	AGVHD III–IV			CGVHD			EXT CGVHD		
	HR	CI	*p*	HR	CI	*p*	HR	CI	*p*
MRD negative at transplant	0.65	0.41–1.03	0.06	0.87	0.67–1.12	0.26	0.57	0.40–0.81	0.00
Age (per 10 years)	1.00	0.83–1.21	0.99	1.02	0.92–1.14	0.64	1.00	0.87–1.16	0.99
RIC vs. MAC	1.22	0.74–2.01	0.44	1.04	0.79–1.35	0.80	0.94	0.65–1.37	0.76
Good risk cytogenetics vs. all others	0.96	0.57–1.62	0.89	1.09	0.77–1.31	0.95	1.02	0.71–1.47	0.91
Time from diagnosis to transplant	1.00	0.99–1.01	0.51	1.00	0.10–1.09	0.27	1.06	0.10–1.01	0.15
VUD vs. MSD	1.65	0.98–2.77	0.06	1.15	0.88–1.50	0.32	1.71	1.16–2.53	0.01
Female donor to male	1.12	0.63–2.10	0.70	1.25	0.92–1.70	0.16	1.45	0.95–2.22	0.08
In vivo TCD	0.46	0.28–0.77	0.00	0.43	0.33–0.56	<10^−5^	0.18	0.12–0.27	<10^−5^
Patient CMV positive	1.06	0.630–1.79	0.83	0.96	0.73–1.24	0.72	1.41	0.96–2.09	0.08
Donor CMV positive	1.52	0.92–2.520	0.10	1.27	0.97–1.64	0.08	1.14	0.78–1.67	0.50
Transplant center			0.36			0.10			0.12

*cGVHD* chronic graft vs. host disease, *aGVHD* acute graft vs. host disease, *CMV* cytomegalovirus, *GRFS* graft vs. host disease and relapse-free survival, *GVHD* graft vs. host disease, *LFS* leukemia-free survival, *MAC* myeloablative conditioning, *MRD* measurable residual disease, *MSD* matched sibling donor, *NRM* non-relapse mortality, *OS* overall survival, *RIC* reduced-intensity conditioning, *TCD* T cell depletion, *MUD* volunteer unrelated donor.

### Analysis of MRD POS vs. MRD NEG in MSD or MUD

Finally, a subgroup analysis was performed according to the donor type, MSD or MUD, in univariate (Supplementary Table 5) and then Cox regression analysis (Supplementary Tables 6, 7).

When an MSD was used, the significant benefits of MRD negative status were maintained for relapse (HR 0.45 CI 0.31–0.65 *P* < 0.001), LFS (HR 0.65 CI 0.48–0.89 *P* = 0.006), GRFS (HR 0.58 CI 0.44–0.76 *P* < 0.001) and extensive cGVHD (HR 0.43 CI 0.26–0.71 *P* < 0.001) but OS was similar to patients with MRD POS status (Supplementary Table 6). As expected, NRM increased with age and was reduced by the use of RIC HCT, while TCD improved GRFS and cGVHD rates, but otherwise patient age, conditioning intensity, cytogenetic status at diagnosis, and TCD made no significant impact on transplant outcomes. Time to transplant from original diagnosis maintained its predictive effect on OS, LFS, NRM, and relapse (Supplementary Table 6). Interestingly, CMV seropositive patients and donors were associated with excess rates of extensive cGVHD and aGVHD grades III–IV, respectively.

The use of an HLA 10/10 MUD appeared to compensate for the presence of MRD since transplant outcomes were similar in MRD POS vs. MRD NEG recipients (Supplementary Table 7). Increasing age was linked to increased NRM while good risk cytogenetics predicted superior OS, LFS, and relapse rates. Relapse rates and LFS were beneficially related to long periods from diagnosis to transplant. Conditioning intensity had no apparent effect on transplant outcomes. The use of TCD reduced all forms of GVHD and improved GRFS but at the expense of OS, probably reflecting trends towards increased relapse and worse LFS. Patients and donors who were CMV positive were associated with excess cGVHD and NRM, respectively.

## Discussion

We report the first large series of transplant outcomes in 1042 adults with AML in CR2 with an established MRD status at transplant. These patients were transplanted in multiple centers, mostly European, who reported details of transplants to the EBMT. Of note, the MSD and MUD groups were balanced except for cytogenetic risk (more good risk in the MSD group, conditioning (more TBI in MSD), and as expected for GVHD prevention.

The Seattle group showed that MRD status prior to HCT was predictive of outcome in 70 patients with AML CR2^31^. These patients had a median age of 42.3 (range 2.1–72.6) years and all were treated with MAC HCT plus, in some cases, in vivo T cell depletion with anti-thymocyte globulin. The EBMT cohort differs from North American subjects by excluding children and by including RIC, as well as MAC HCTs, and collecting GRFS status. MRD status was assessed by multi-parameter flow cytometry immunophenotyping and/or by PCR-based assays according to center preference^47^. Unfortunately, we did not have individual information on the method used that would allow us to compare the outcome between PCR and flow cytometry. Despite this limitation, we found that MRD negative patients had a substantially reduced risk of relapse at 24.1% compared to 39.8% in MRD positive recipients, and this translated into a superior LFS and GRFS (Table 2 and Supplementary Table 4). However, MRD status had no independent impact on OS although MRD negative status was associated with a trend to increased NRM which may have partially offset the improved LFS (Table 3 and Supplementary Table 4). The largest effects on OS were exerted by the established risk factors of cytogenetic status at diagnosis and the time interval from diagnosis to transplant and these also impacted LFS (Table 3 and Supplementary Table 4). The relatively few patients with adverse risk cytogenetics reflect standard practice to offer transplant in CR1.

The fitness of patients for transplant may be evaluated by a combination of performance status (PS), comorbidity index, and frailty assessment^56–58^. Due to a lack of data we were only able to study the impact of Karnofsky (PS) and found no impact on transplant outcomes in multivariate analysis (Supplementary Table 4). This may possibly reflect a more tailored transplant approach by centers to patients with lower PS.

We also looked at the effect of TCD as this is used extensively in Europe. In this cohort, TCD was associated with increased relapse rates and improved GRFS but no difference in LFS and OS. Increased RI in association with TCD in older patients with AML CR1 MRD NEG status at transplant has also been reported^35^. However, the use of ATG as TCD has not generally been associated with significant increases in relapse risk in other studies performed by the EBMT Acute Leukemia Working Party, although these have been predominantly performed in patients with AML CR1 rather than CR2^47,59,60^. The use of TCD in vivo with anti-thymocyte globulin or Alemtuzumab requires further prospective study due to conflicting results in published studies which vary in disease stage, absolute lymphocyte count at the time of TCD, dose schedule, and associated conditioning regimen^47,61–65^.

We have previously studied the effect of conditioning intensity in AML CR1 and found that MAC HCT was superior to RIC HCT only in patients <50 years who were MRD pos at HCT^35^. Overall, in this cohort, we found no benefit to increased intensity of conditioning. Patients under the median age of 49.4 years had better NRM, LFS, and OS at the expense of higher aGVHD rates than older counterparts in univariate analysis (Supplementary Table 4). In multivariate analysis, however, the only effect of increasing age was to increase NRM. This is in keeping with our previous study of conditioning intensity in HCT AML CR2 where there is no impact on survival in patients under 50 years but an increase in NRM for patients of 50 or more years, particularly following MAC HCT^45^.

The adverse impact of MRD POS status on RI and LFS in the whole group was also seen when patients in receipt of sibling donors were studied but interestingly this effect was not evident in those patients receiving MUD HCT. This sub-group analysis supports the possibility that the use of a volunteer donor confers an enhanced graft vs leukemia effect and may be preferable to a sibling donor in AML CR2 MRD POS, thus contributing to the ongoing debate about the relative merits of sibling vs. volunteer donors^66–68^. The advent of high-resolution HLA typing has reduced NRM in MUD HCT and may allow the graft vs. leukemia effect to be studied in a more homogenous setting in future studies^69^.

While we did not see any impact of MRD status on OS in this study, in contrast to the effects of cytogenetic risk group and time from diagnosis to transplant, we did not have details of post-HCT salvage regimens such as targeted therapy with FLT3 inhibitors or donor lymphocyte infusions (DLI). We also lacked data on the precise methodology and validation criteria of the MRD assays used by each center. Since this was a registry analysis we cannot exclude bias on the part of the centers with respect to the decision to transplant. In addition, it was not possible to ascertain the precise duration of CR1 or the FLT3 and NPM1 status at diagnosis for all patients.

Monitoring of MRD status peri-HCT is rapidly becoming standard clinical practice for patients with AML with consequent recourse to DLI and investigational drugs were available for patients with evidence of MRD. However, international standardization of MRD assays will be key to future insights into the implications of MRD.

## Supplementary information

Supplementary Table 1: Conditioning regimens

Supplementary Table 2 Graft versus Host disease prophylaxis

Supplementary Table 3 Transplant Outcomes

Supplementary Table 4 Univariate analysis of transplant outcomes

Supplementary Table 5 Univariate analysis of transplant outcomes by donor type

Supplementary Table 6 Multivariate analysis of transplant outcomes in recipients of HLA matched sibling donors

Supplementary Table 7 Multivariate analysis of transplant outcomes in recipients of HLA 10/10 matched volunteer unrelated donors
